# Clinicopathological signature of p21-activated kinase 1 in prostate cancer and its regulation of proliferation and autophagy via the mTOR signaling pathway

**DOI:** 10.18632/oncotarget.15124

**Published:** 2017-02-06

**Authors:** Zhanyu Wang, Guojin Jia, Yan Li, Jikai Liu, Jinfang Luo, Jihong Zhang, Guoxiong Xu, Gang Chen

**Affiliations:** ^1^ Department of Urology, Jinshan Hospital, Fudan University, Shanghai 201508, People's Republic of China; ^2^ Department of Pathology, Jinshan Hospital, Fudan University, Shanghai 201508, People's Republic of China; ^3^ Center Laboratory, Jinshan Hospital, Fudan University, Shanghai 201508, People's Republic of China

**Keywords:** PAK1, prostate cancer, mTOR signaling, rapamycin, MYH1485

## Abstract

Prostate cancer (PCa) is one of the most common malignant tumors in men. The etiology and pathogenesis of PCa remain unclear. P21-activated kinase 1 (PAK1) is a member of a family of serine/threonine kinases and regulates cell growth and contributes to tumor invasion and metastasis. However, the association of PAK1 with PCa tumorigenesis and in particular with cell autophagy remains unknown. We found that the positive expression of PAK1 was significantly increased in PCa patients compared with BPH patients (*P* < 0.05). The expression of PAK1, p-PAK1 and LC3B1 in DU145 was increased by the activator of mTOR MYH1485. The expression of PAK1, p-PAK1, mTOR and Beclin1 decreased in PAK1-shRNA expressing DU145 cell. Knocking down of PAK1 inhibited DU145 cell growth, invasion and migration *in vitro*, and inhibited tumor growth *in vivo*. Our study demonstrated that PAK1 is upregulated in PCa and regulated by the mTOR signaling pathway and contributes to tumor autophagy. Thus, PAK1 may be a potential tumor marker and therapeutic target of PCa.

## INTRODUCTION

Prostate cancer (PCa) is the most frequently diagnosed cancer accounting for 1 in 5 new diagnosis with an estimated 180,890 new diagnoses and the second leading cause of cancer deaths in men with an estimated 26,120 mortalities in the United States in 2016 [1]. As one of the 10 most common cancers considered in the temporal trend analyses for men in China [2], prostate cancer incidence rates from 2000 to 2011 increased with an upward trend in age-standardized mortality rates. The factors driving the increase in PCa are not completely understood. The therapeutic side effects of PCa can be serious [3] and no curative treatment exists when PCa become androgen resistant. Prostate specific antigen (PSA), being organ-specific, is produced mainly by the epithelial cells of the prostate. The American Food and Drug Administration (FDA) approved PSA for monitoring disease progression in 1986, and endorsed it for PCa screening among men aged ≥ 50 years [4, 5]. But PSA screening remains controversial because of the benefit of early detection and the false-positive results. Therefore, to improve the survival and quality of life of patients with PCa, it is urgent to identify therapeutic and diagnostic biomarkers and to explore the underlying mechanisms of tumor progression.

As one of the first classes of Rho-GTPases regulated kinases to be identified [6], p21-activated kinases (PAKs) are a family of evolutionarily conserved group of serine-threonine kinases that are classified into two groups based on the structure and functional features: group I (PAK1-3) and group II (PAK4-6) [7]. PAK1 is involved in a variety of biological activities, including cellular functions, steroid receptor signaling, gene transcription and oncogenic transformation [8]. Even though the deregulation of PAK1 is closely associated with various human diseases, and PAK1 plays a crucial role in tumor genesis, progression, invasion and metastasis in several types of human tumor [9–11]. Several PAK inhibitors have been developed for use as biological probes and therapeutic agents [12], and ATP-competitive inhibitors may have relatively poor selectivity for the similarity between the ATP-binding pockets of kinases of the same family, PAK-selective ATP-competitive inhibitors have been identified [13, 14]. But the contribution of PAK1 to cancer autophagy, prognosis, therapeutic target and PAK1 signaling pathway remains limited.

Rapamycin is a macrolide antibiotic from Streptomyces hygroscopicus that has been approved by the US FDA as an immunosuppressant and is commonly used to prevent rejection in organ or bone-marrow transplant patients. Rapamycin [15] inhibits the mammalian target of rapamycin (mTOR), a serine/threonine kinase often upregulated in malignant cells and played a key role in tumor development and progression.

MHY1485 is synthesized based on its morpholino triazine structure that is known to bind mTOR, and was selected based on the results of the ratio of LC3BII/LC3BI, and LC3II protein largely accumulated and autophagosomes enlarged by the treatment of MHY1485, which is another regulator of autophagy and induces mTOR activity [16].

## RESULTS

### PAK1 was associated with the clinicopathological features of patients with PCa and BPH

A total of 113 patients were included in this study with a median follow-up time ranging from 10 to 102 months (38.22 ± 24.56 months). Ten of the 41 PCa patients died during the follow-up period, and only one of the 24 BPH patients died 10 months after surgery during the follow-up period. It was statistically significant that PSA and Alkali phosphatase (AKP) were higher in PCa than BPH, and the larger prostate size, more tubercle existence, central sulcus disappearance and scleroid texture, and more abnormal echo of ultrasonography, abnormal lesion of Computed tomography and abnormal signal of Magnetic resonance imaging could be found in PCa (Table 1). The PSA and AKP of metastasis PCa patients were higher (Table 2).

**Table 1:** Association of PAK1 expression with clinicopathological features of patients with PCa and BPH patients, and indexes

	Pca				BPH				*P*-value^c^
	positive	negative	total	*P*-value^a^	positive	negative	total	*P*-value^b^	
**Age**				0.127				0.336	0.058
< 70	11	9	20		2	16	18		
≥ 70	39	14	53		5	17	22		
**PSA**				0.001*					0.000*
< 50	14	16	30		7	33	40		
≥ 50	36	7	43		0	0	0		
**Haematoglobin**				0.515				0.041*	0.105
< 130	28	11	39		5	10	15		
≥ 130	22	12	34		2	23	25		
**AKP**				0.980					0.001*
< 150	39	18	57		7	33	40		
≥ 150	11	5	16		0	0	0		
**Neutrophil/achroacyte**				0.530				0.787	0.650
< 2.8	30	12	42		4	17	21		
≥ 2.8	20	11	31		3	16	19		
**Complaint**									
difficulty of urination	36	17	53		5	24	29		
hematuria	4	0	4		0	7	7		
frequency, urgency, dysuria	4	4	8		2	1	3		
health examination	6	2	8		0	1	1		
**DRE**									
Size				1.000				0.667	0.023*
1–2	35	15	50		6	30	36		
3–4	14	6	20		1	3	4		
texture				0.003*				0.020*	0.000*
flexible	3	7	10		5	32	37		
scleroid	46	14	60		2	1	3		
tubercle				0.083					0.000*
yes	25	6	31		0	0	0		
no	24	15	39		7	33	40		
central sulcus				0.483					0.008*
yes	40	19	59		7	33	40		
no	9	2	11		0	0	0		
no examination	1	2	3		0	0			
**Hypertension**				0.884				0.388	0.677
yes	27	12	39		3	20	23		
no	23	11	34		4	13	17		
**Ultrasonography**				0.461					0.027*
abnormal echo	8	5	13		0	1	1		
no abnormal echo	15	5	20		4	9	13		
**CT**				0.603					0.000*
abnormal lesion	23	8	31		0	0	0		
no abnormal lesion	3	2	5		2	13	15		
**MRI**				0.119					0.000*
abnormal signal	24	7	31		0	0	0		
no abnormal signal	2	3	5		1	3	4		
**Gleason score**				0.924					
≤ 6	12	5	17						
7	27	12	39						
≥ 8	11	6	17						

AKP, Alkali phosphatase; DRE, digital rectal examination; CT, Computed tomography, MRI, Magnetic resonance imaging *P*-value^a^, PAK1 expression in PCa; *P*-value^b^, PAK1 expression in BPH; *P*-value^c^, PAK1 expression between PCa and BPH.

*P < 0.05.

**Table 2:** Clinicopathological indexes of no metastasis PCa and metastasis PCa

	no metastasis	metastasis	*P*-value
**Gleason score**			0.083
≤ 6	15	2	
7	23	16	
≥ 8	10	7	
**Age (years)**			0.457
< 70	12	8	
≥ 70	36	17	
**PSA (ng/ml)**			0.011*
≤ 20	7	3	
20–100	29	7	
> 100	12	15	
**Haematoglobin (g/L)**			
< 130	21	18	0.022*
≥ 130	27	7	
**AKP (IU/L)**			0.000*
< 150	46	16	
≥ 150	2	9	
**Neutrophil/achroacyte**			0.254
< 2.8	26	17	
≥ 2.8	22	8	
**DRE**			
size			0.937
1–2	33	17	
3–4	13	7	
texture			0.681
flexible	6	4	
scleroid	40	20	
tubercle			0.319
yes	23	9	
no	23	15	
central sulcus			0.395
existing	40	19	
disappeared	6	5	
no examination	2	1	
**Complaint**			0.853
difficulty of urination	35	18	
difficulty of urination AND urinary retention	2	4	
difficulty of urination AND hematuria	2	1	
hematuria	2	2	
frequency, urgency, dysuria	5	3	
health examination	6	2	
**Hypertension**			0.072
yes	22	17	
no	26	8	

* *P* < 0.05.

The Chi-squared test was used to evaluate the correlation of PAK1 expression with age, PSA, pathology, Gleason score, DRE, metastasis, haematoglobin, AKP, Neutrophil/achroacyte, Complaint and hypertension, et al. The expression of PAK1 in PCa patients was found to have statistically significant correlations with PSA and prostate texture, while the expression of PAK1 in BPH patients was found to have statistically significant correlations with haematoglobin and prostate texture (Table 1).

### Immunohistochemistry staining of PCa and BPH tissues

To examine the expression of PAK1, p-PAK1, mTOR and p-mTOR in PCa and BPH tissues derived from patients, IHC staining was applied. A total of 73 PCa and 40 BPH patients were included in this study. The expression of PAK1, p-PAK1, mTOR and p-mTOR protein was typically cytoplasmic. PAK1 and p-PAK1 was overexpressed in human PCa tissue compared with those in BPH tissue, whereas mTOR and p-mTOR did not be demonstrated obviously diversity pattern between PCa and BPH tissue.

**Figure 1:**
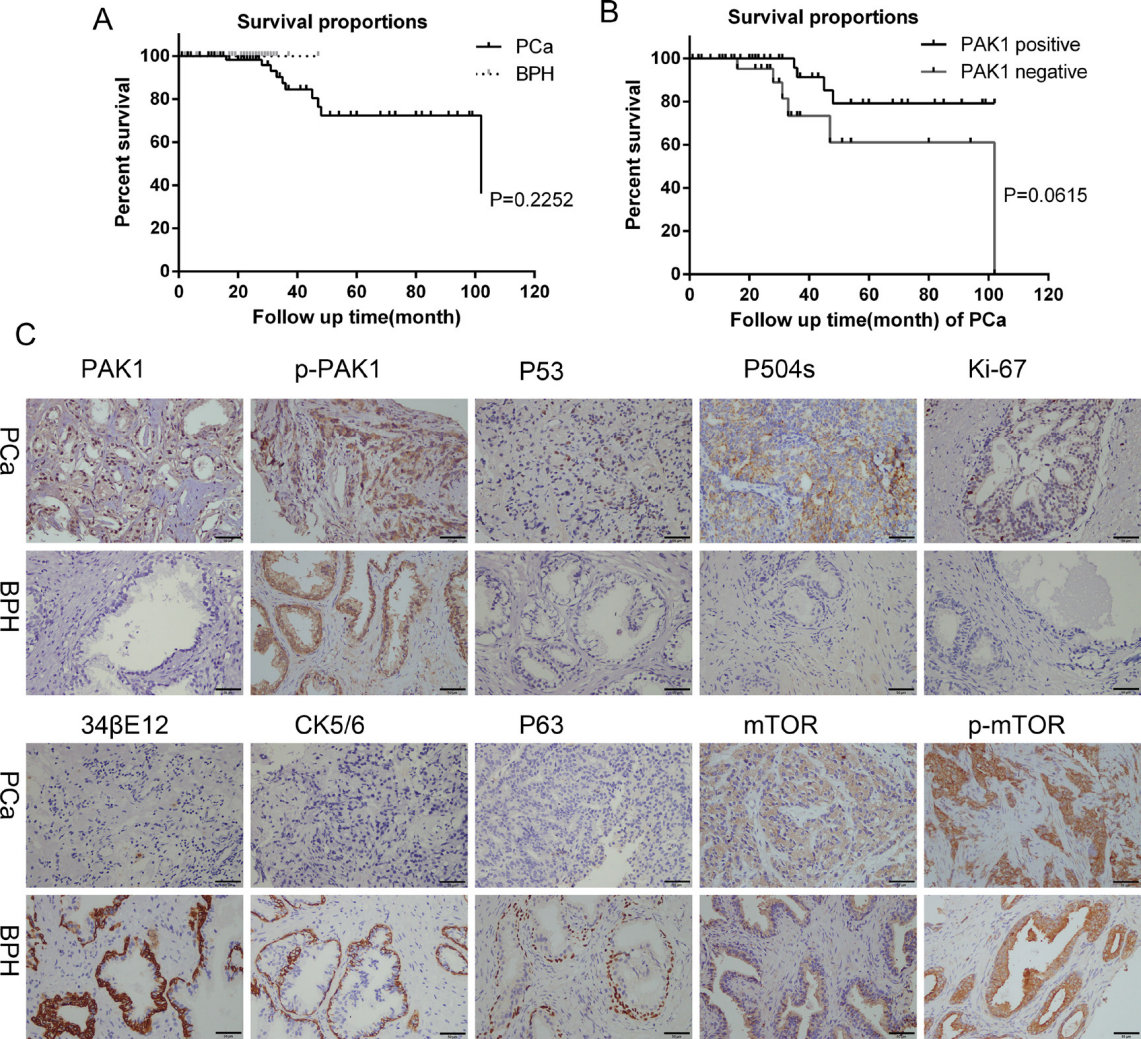
Survival curves, immunohistochemical staining for PAK1, p-PAK1, P53, P504s, Ki-67, 34βE12, CK5/6, P63, mTOR and p-mTOR protein expression of PCa and BPH patients tissues (**A**) Survival curves of PCa and BPH patients. (**B**) Survival curves of PAK1 positive and negative of PCa patients. (**C**) Higher level of PAK1, P53, P504s, Ki-67 expression was observed in PCa tissues than in BPH, while higher level of 34βE12, CK5/6 and P63 expression was observed in BPH than in PCa. P-PAK expression was positive in PCa and BPH. There was no difference in mTOR and p-mTOR expression between PCa and BPH.

The expression of 34βE12, P63 and CK5/6 was decreased to more negative levels in human PCa tissue compared with BPH, while the expression p53 overexpressed in human PCa. The positive expression of P504s and Ki-67 was higher in PCa tissue than BPH. (Figure 1, Table 3)

**Table 3:** Immunohistochemistry of human PCa and BPH tissues

	PCa		BPH		
	Positive	Negtive	Positive	Negtive	*P*-value
PAK1	50	23	7	33	0.000*
p-PAK1	70	1	19	4	0.003*
mTOR	17	10	10	5	0.81
p-mTOR	26	0	14	1	0.183
PSA	61	0	21	0	
PAP	52	0	21	0	
34βE12	1	64	23	0	0.000*
P63	0	61	21	0	0.000*
P53	24	21	2	12	0.010*
CK5/6	0	24	14	3	0.000*
P504S	65	0	0	23	0.000*
Ki-67	21	27	0	13	0.003*
AR	23	2	13	2	0.586
CK	31	2	11	0	0.403
CK20	2	14	0	6	
CK7	1	11	3	0	
Vimentin	1	15	1	0	
SMA	5	9	4	1	

* *P* < 0.05.

### Expression of PAK1 mRNA and protein in patients with PCa and prostatic epithelial cells

**Figure 2:**
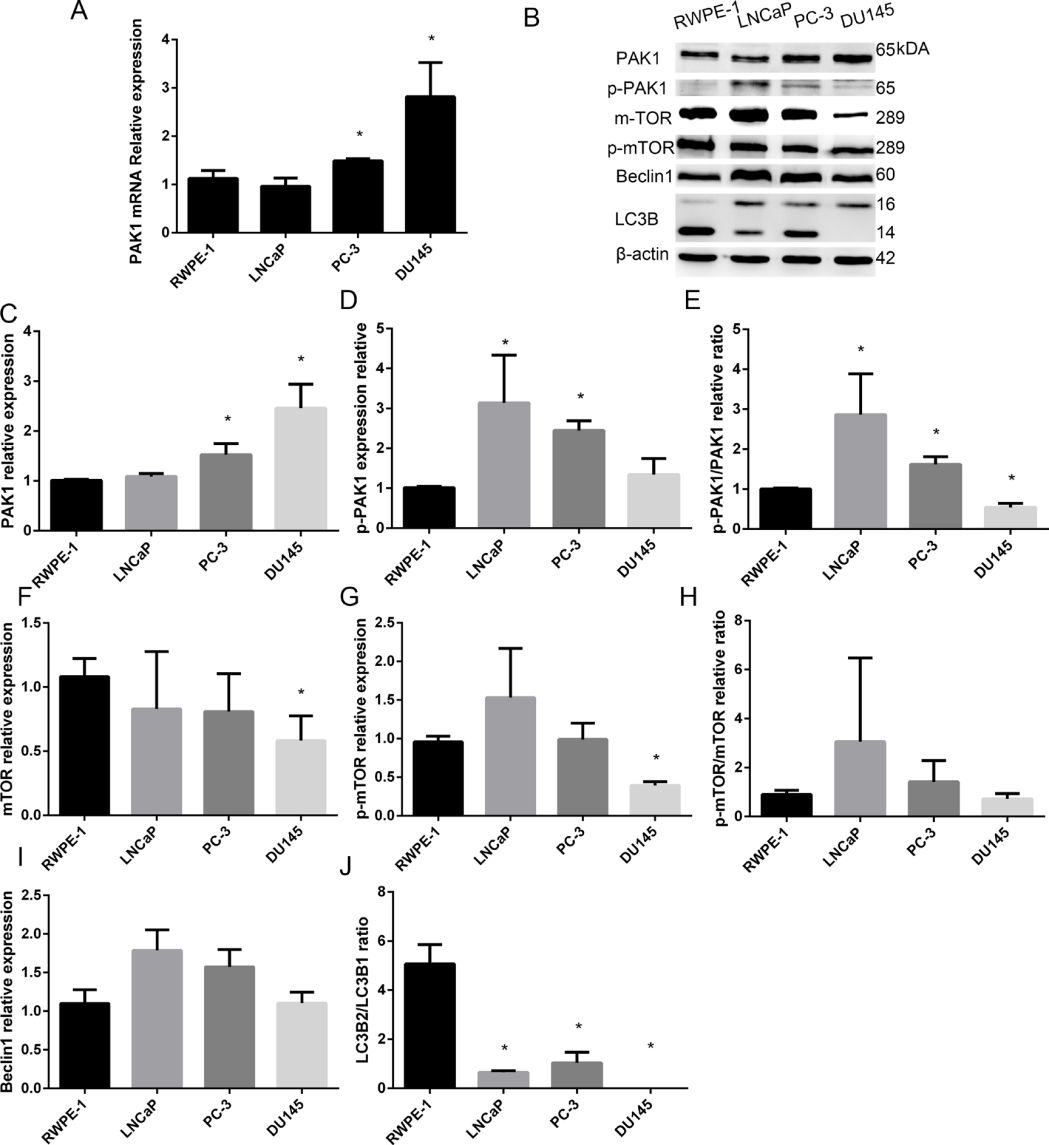
Expression of PAK1 mRNA and protein in patients with PCa and prostatic epithelial cells (**A**) PAK1 mRNA was higher in the lines DU145 than PC-3, LNCaP and RWPE-1, and the expression of PAK1 in PC-3 was higher than LNCaP and RWPE-1. (**B**) Western blot (**C**) PAK1 protein expression was significantly higher in DU145 than PC-3, LNCaP and RWPE-1, and PAK1 expression was higher in PC-3 than LNCaP and RWPE-1. (**D**) p-PAK1 expression in LNCaP and PC-3 was higher than in DU145 and RWPE-1. (**E**) p-PAK1/PAK1 ratio in LNCaP and PC-3 was higher than in RWPE-1, and the ratio was lower in DU145 than in RWPE-1. (**F**) mTOR expression in DU145 was lower than RWPE-1 and LNCaP. (**G**) p-mTOR in DU145 was lower than RWPE-1, LNCaP and PC-3. (**H**) p-Mtor/Mtor ratio. (**I**) Beclin1 expression in DU145 was lower than in LNCaP. (**J**) LC3B2/LC3B1 ratio in DU145 was lower than in RWPE-1, LNCaP and PC-3, meanwhile, the ratio in LNCaP and PC-3 was significantly less than RWPE-1. *P < 0.05.

To verify the expression of the PAK1 gene in PCa, we assessed the relative abundance of PAK1 in three human PCa cell (DU145, LNCaP and PC-3) and human prostatic epithelial cell (RWPE-1) lines using quantitative real-time PCR. DU145 and PC-3 cells are considered androgen-independent human PCa cell lines, whereas the LNCaP cells are androgen-dependent human PCa cell lines. The PCR results indicated that the expression of PAK1 was higher in the lines DU145 than PC-3, LNCaP and RWPE-1, and the expression of PAK1 was higher in the lines PC-3 than LNCaP and RWPE-1 (Figure 2).

### Expression of PAK1, p-PAK1, mTOR, p-mTOR, Beclin1 and LC3B in PCa and prostatic epithelial cells

Based on the PCR results, we evaluated the expression of PAK1 protein in the four prostate cell lines (DU145, PC-3, LNCaP and RWPE-1) using western blot analysis.

As shown in Figure 2, Western blot analysis showed that PAK1 expression was significantly higher in DU145 than PC-3, LNCaP and RWPE-1, and PAK1 expression was higher in PC-3 than LNCaP and RWPE-1, while p-PAK1 expression in LNCaP and PC-3 was higher than in DU145 and RWPE-1. MTOR expression in DU145 was lower than RWPE-1 and LNCaP, while p-mTOR in DU145 was lower than RWPE-1, LNCaP and PC-3. Beclin1 expression in DU145 was lower than in LNCaP. LC3B2/LC3B1 ratio in DU145 was lower than in RWPE-1, LNCaP and PC-3, meanwhile, the ratio in LNCaP and PC-3 was significantly less than RWPE-1.

### Rapamycin and MHY1485 treatment in DU145, LNCaP and RWPE-1 cell lines

The states of the DU145, LNCaP and RWPE-1 cells were almost identical before being treated with rapamycin and MYH1485, respectively. Different concentrations of rapamycin inhibited cells proliferation unequally, and low concentrations (1, 10 and 100 nmol/L) of rapamycin didn't inhibit cells proliferation obviously after 48 h, while rapamycin at the concentration of 1000 nmol/L inhibited DU145, LNCaP and RWPE-1 cell lines apparently, almost to no invisible levels after 24 h. Different concentrations of MYH1485 promoted cells proliferation unequally, and low concentrations (0.1, 1 and 10 μmol) of MYH1485 didn't promote cells proliferation obviously after 48 h, while the concentration 100 μmol promoted DU145, LNCaP and RWPE-1 cell lines apparently after 12 h, the cells were proliferated to macroscopic yellowish thin-layer with 100 μmol MYH1485 after 12 h, and the majority of cells died and few cells survived after 24 h.

**Figure 3:**
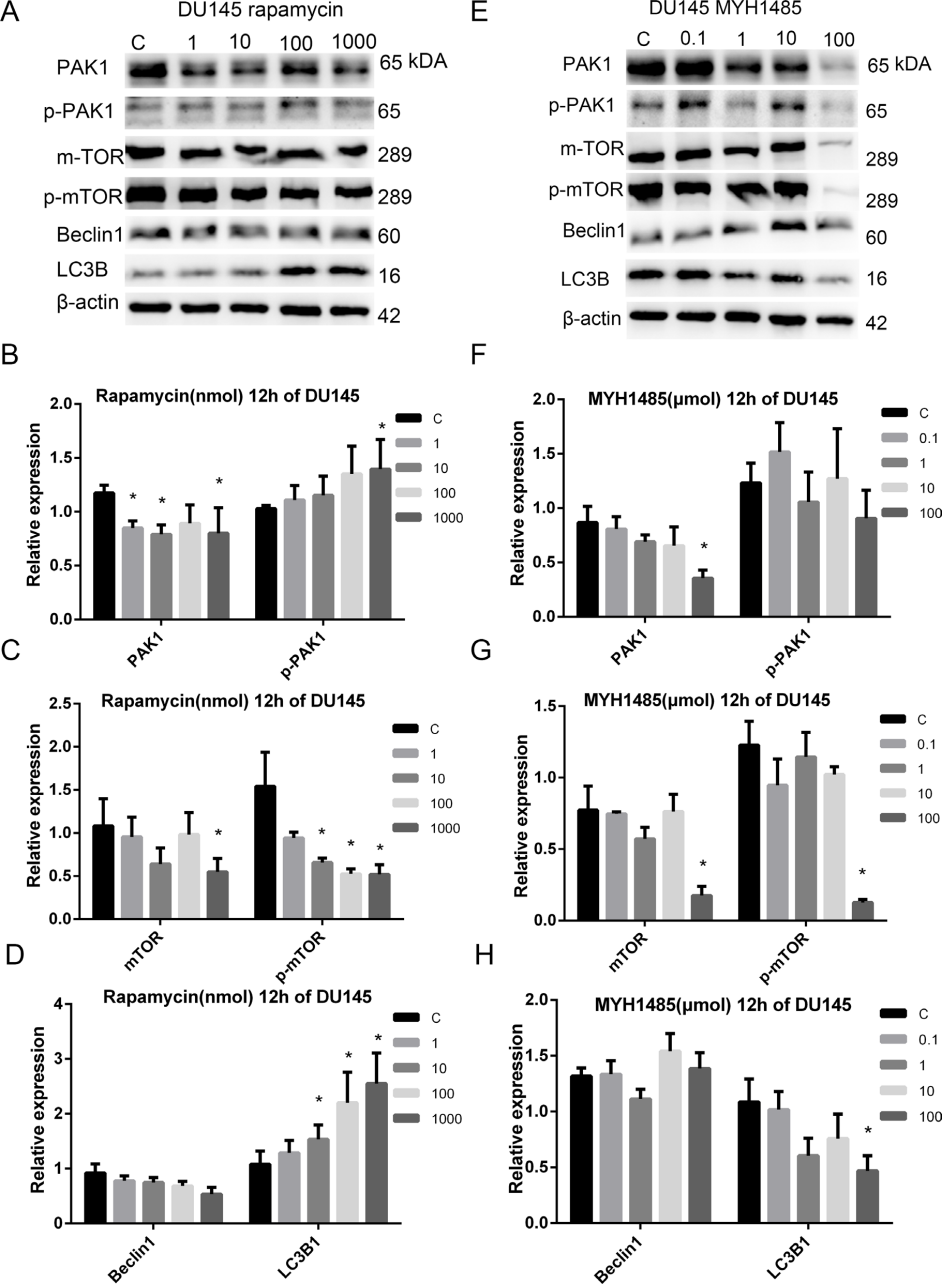
Rapamycin and MHY1485 treatment of DU145 cell lines with 12 h. (**A**) Rapamycin treatment of DU145 with 12 h. (**B**) PAK1 expression decreased by rapamycin with 1, 10 and 1000 nmol than the control. p-PAK1 expression increased by rapamycin with 1000 nmol. (**C**) mTOR expression decreased by rapamycin with 1000 nmol. p-mTOR expression decreased by rapamycin with 10, 100 and 1000 nmol. (**D**) LC3B1 expression increased by rapamycin with 10, 100 and 1000 nmol. (**E**) MYH1485 treatment of DU145 with 12 h. (**F**) PAK1 expression decreased by MYH1485 with 1000 nmol. (**G**) mTOR and p-mTOR expression decreased by MYH1485 with 100 umol. (**H**) LC3B1 expression decreased by MYH1485 with 100 umol. *P < 0.05.

As shown in Figure 3, treatment of DU145 after 12 h, 2 μl DMSO in 2 ml medium was used as a control when detecting rapamycin treatment while 1 μl DMSO in that when detecting MYH1485 treatment. PAK1 expression decreased with 1, 10 and 1000 nmol rapamycin, and p-mTOR expression decreased with 10, 100 and 1000 nmol rapamycin, while LC3B1 expression obviously increased with 10, 100 and 1000 nmol rapamycin. MTOR and p-PAK1 expression decreased with 1000 nmol rapamycin. PAK1, LC3B1, mTOR, and p-mTOR expression decreased with 1000 μmol MYH1485.

The concentration 10 nmol rapamycin and 0.1 μmol MYH1485 was selected to study DU145, LNCaP and RWPE-1 cells with 1, 6, 12, 24 and 48 h, respectively. DMSO 1.5 μl in 2 ml medium was the control, cells were cultured 24 h after DMSO addition.

**Figure 4:**
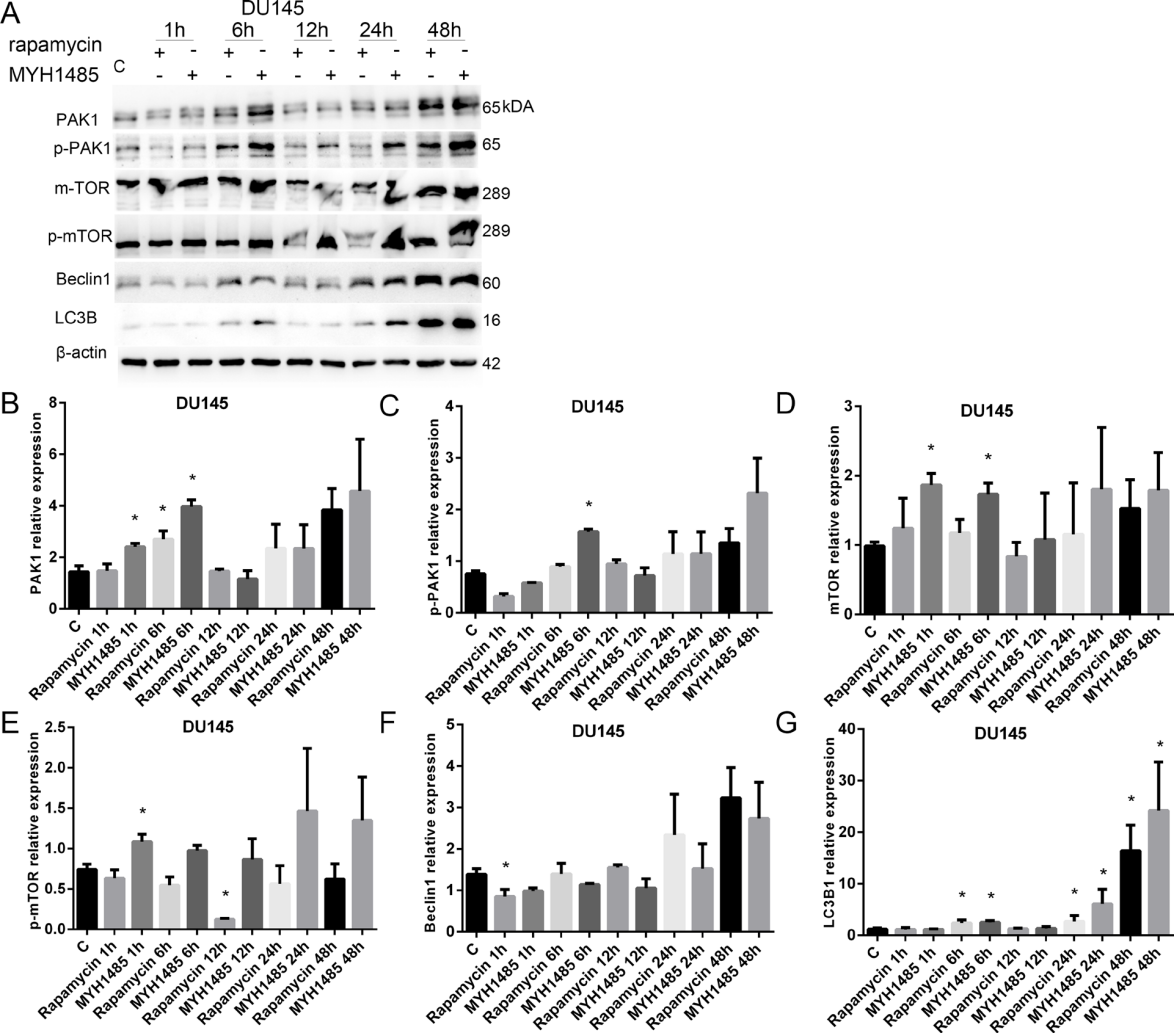
Rapamycin and MHY1485 treatment of DU145 cell lines (**A**) Western blot. (**B**) PAK1 expression increased by 6 h rapamycin and 1 and 6 h MYH1485 than control. (**C**) p-PAK1 expression increased by 6 h MYH1485. (**D**) mTOR expression increased by 1 and 6 h MYH1485. (**E**) p-mTOR expression increased by 1 h MYH1485 and decreased by 12 h rapamycin. (**F**) Beclin1 expression decreased by 1 h rapamycin. (**G**) LC3B1 expression increased by 6, 24 and 48 h of rapamycin or myh1485. *P < 0.05.

As shown in Figure 4, in DU145, PAK1 expression increased with 6 h of rapamycin, and 1 and 6 h of MYH1485. p-PAK1 expression increased with 6 h of MYH1485, mTOR expression increased with 1 and 6 h of MYH1485, and p-mTOR expression increased with 1 h of MYH1485 while decreased 12 h of rapamycin. Beclin1 expression decreased with 1 h of rapamycin. LC3B1 expression increased with 6, 12 and 24 h of rapamycin or MYH1485.

**Figure 5:**
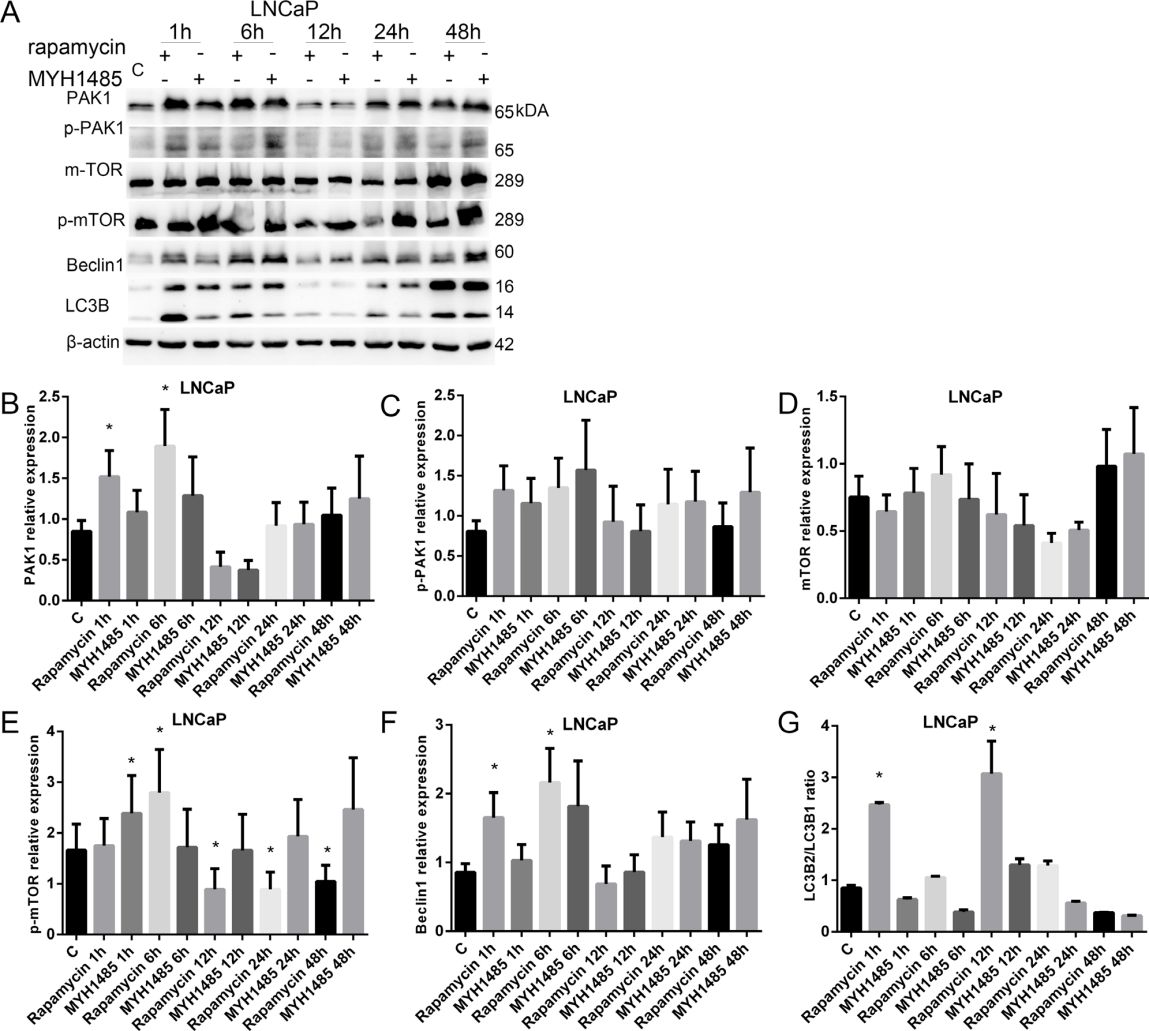
Rapamycin and MHY1485 treatment of LNCap cell lines (**A**) Western blot. (**B**) PAK1 expression increased by 1 and 6 h rapamycin. (**C**) p-PAK1 expression. (**D**) mTOR expression. (**E**) p-mTOR expression increased by 1 h MYH1485 and 6 h rapamycin, but decreased by 12, 24 and 48 h rapamycin. (**F**) Beclin1 expression increased by 1 and 6 h rapamycin. (**G**) LC3B2/LC3B1 ratio increased by 1 and 12 h rapamycin. *P < 0.05.

As shown in Figure 5, in LNCaP, PAK1 and Becclin1 increased with 1 and 6 h of rapamycin. p-mTOR increased with 1 h of MYH1485 and 6 h of rapamycin, but decreased with 12, 24 and 48 h of rapamycin. LC3B2/LC3B1 ratio increased with 1 and 12 h of rapamycin treatment.

**Figure 6:**
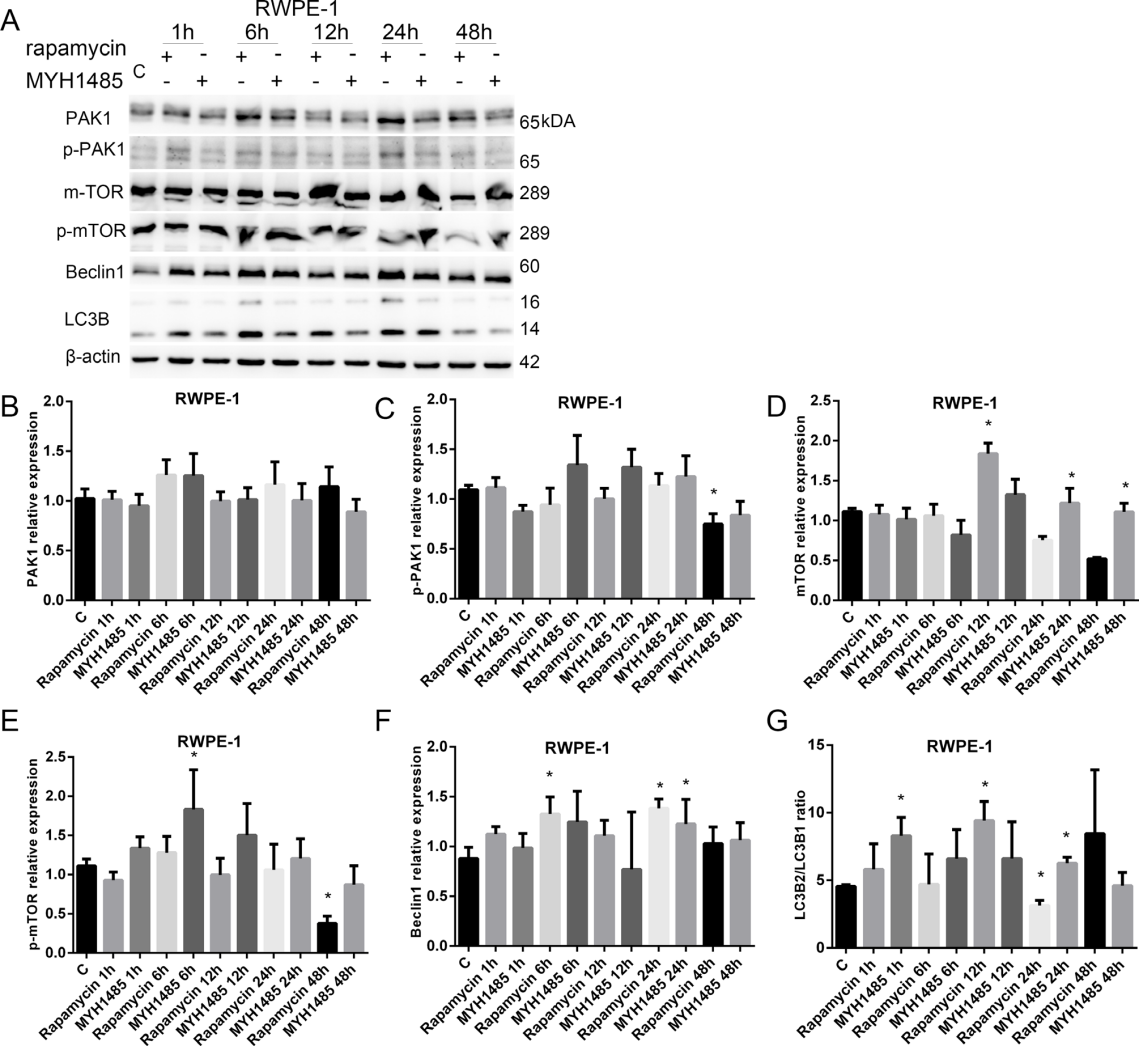
Rapamycin and MHY1485 treatment of RWPE-1 cell lines (**A**) Western blot. (**B**) PAK1 expression. (**C**) p-PAK1 expression decreased by 48 h rapamycin. (**D**) mTOR expression increased by 12 h rapamycin and 24 and 48 h MYH1485. (**E**) p-mTOR expression increased by 6 h MYH1485 and decreased by 48 h rapamycin. (**F**) Beclin1 expression increased by 6 and 24 h rapamycin and 24 h MYH1485. (**G**) LC3B2/LC3B1 ratio increased by 1 and 24 h MYH1485 and 12 h rapamycin, but decreased by 24 h rapamycin. *P < 0.05.

As shown in Figure 6, in RWPE-1, p-PAK1 decreased with 48 h of rapamycin. mTOR increased with 12 h of rapamycin and 24 and 48 h of MYH1485. p-mTOR increased with 6 h of MYH1485, but decreased with 48 h of rapamycin. Beclin1 increased with 24 h of MYH1485 and 6 and 24 h of rapamycin. LC3B2/LC3B1 ratio increased with 12 h of rapamycin and 1 and 24 h of MYH1485 treatment, and decreased with 24 h of rapamycin.

### PAK1 was involved in DU145 cell proliferation, migration, and invasion

**Figure 7:**
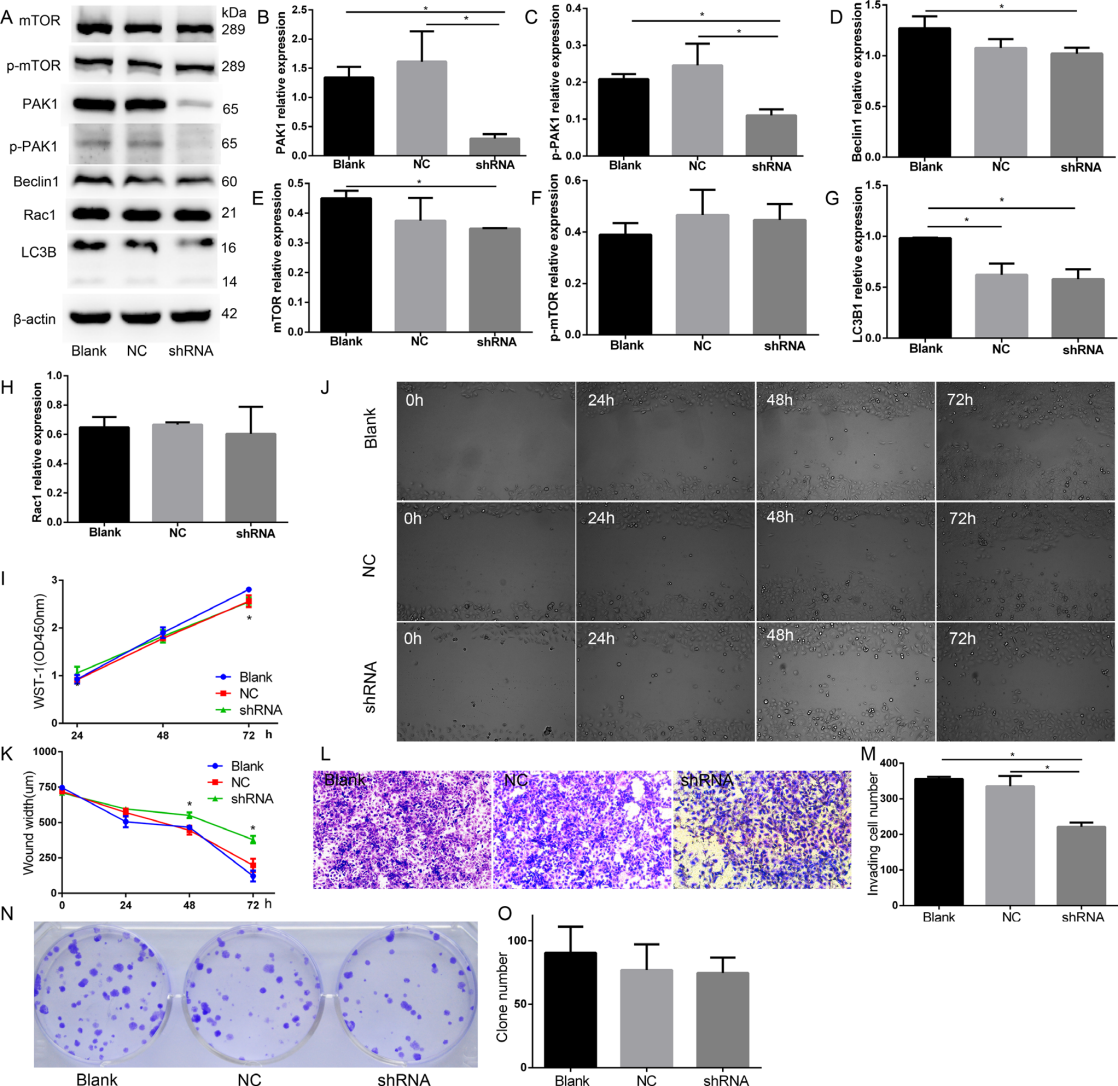
PAK1 was involved in DU145 cell proliferation, migration, and invasion (**A**) Western blot. (**B**) PAK1 expression decreased in PAK1-shRNA expressing cells than Blank and NC. (**C**) p-PAK1 expression decreased in PAK1-shRNA expressing cells than Blank and NC. (**D**) Beclin1 expression decreased in PAK1-shRNA expressing cells than Blank. (**E**) mTOR expression decreased in in PAK1-shRNA expressing cells than Blank. (**F**) p-mTOR relative expression. (**G**) LC3B1 relative expression. (**H**) Rac1 relative expression. (**I**) WST-1 assay, the decrease of cell growth in PAK1-shRNA expressing cells was confirmed on 72 h after seeding, compared with the blank. (**J**, **K**) A wound-healing assay. Knocking down of PAK1 inhibited cell migration compared with the blank and NC on 48 and 72 h after seeding. (**L**, **M**) Cell invasion assay. Knocking down of PAK1 resulted in a decrease of invasion in DU145 cells after culture for 48 h. (**N**, **O**) Colony formation. *P < 0.05.

To determine the functional effects of PAK1 on the biological behaviors of DU145 cells, knocking down of PAK1 using shRNA approach was applied. We generated PAK1-shRNA stably expressing cells and found that no morphology of PAK1-shRNA expressing cells (shRNA) was changed after five passages. Fluorescent image showed that more than 95% cells at passage 5 were GFP-positive as the PAK1-shRNA construct contains GFP, and the expression of PAK1 was significantly decreased both at mRNA and protein levels detected by quantitative RT-PCR and Western blot compared with the normal DU145 cells (Blank) and negative control (NC), respectively (Figure 7A, 7B), and p-PAK1 expression decreased in PAK1-shRNA expressing cells than Blank and NC, Beclin1 and mTOR expression decreased in p-mTOR, LC3B1 and Rac1 expression in PAK1-shRNA expressing cells compared with NC cells (Figure 7F–7H). Than Blank (Figure 7C–7E).To examine cell proliferation by WST-1 assay, the decrease of cell growth in PAK1-shRNA expressing cells was confirmed on day 3 after seeding compared with the Blank (Figure 7I). A wound-healing assay was performed in DU145 cells to evaluate the effect of PAK1 on cell migration, and the suppression of PAK1 expression significantly inhibited DU145 cell migration compared with the blank and NC. We found that knocking down of PAK1 resulted in a decrease of invasion in DU145 cells after culture for 48 h (Figure 7L, 7M). The colony formation ability of PAK1-shRNA DU145 cells tended to decrease when comparing with the normal DU145 cells in the colony formation assay (Figure 7N, 7O).

### Knockdown of PAK1 by shRNA inhibits the growth of DU145 cells *in vivo*

**Figure 8:**
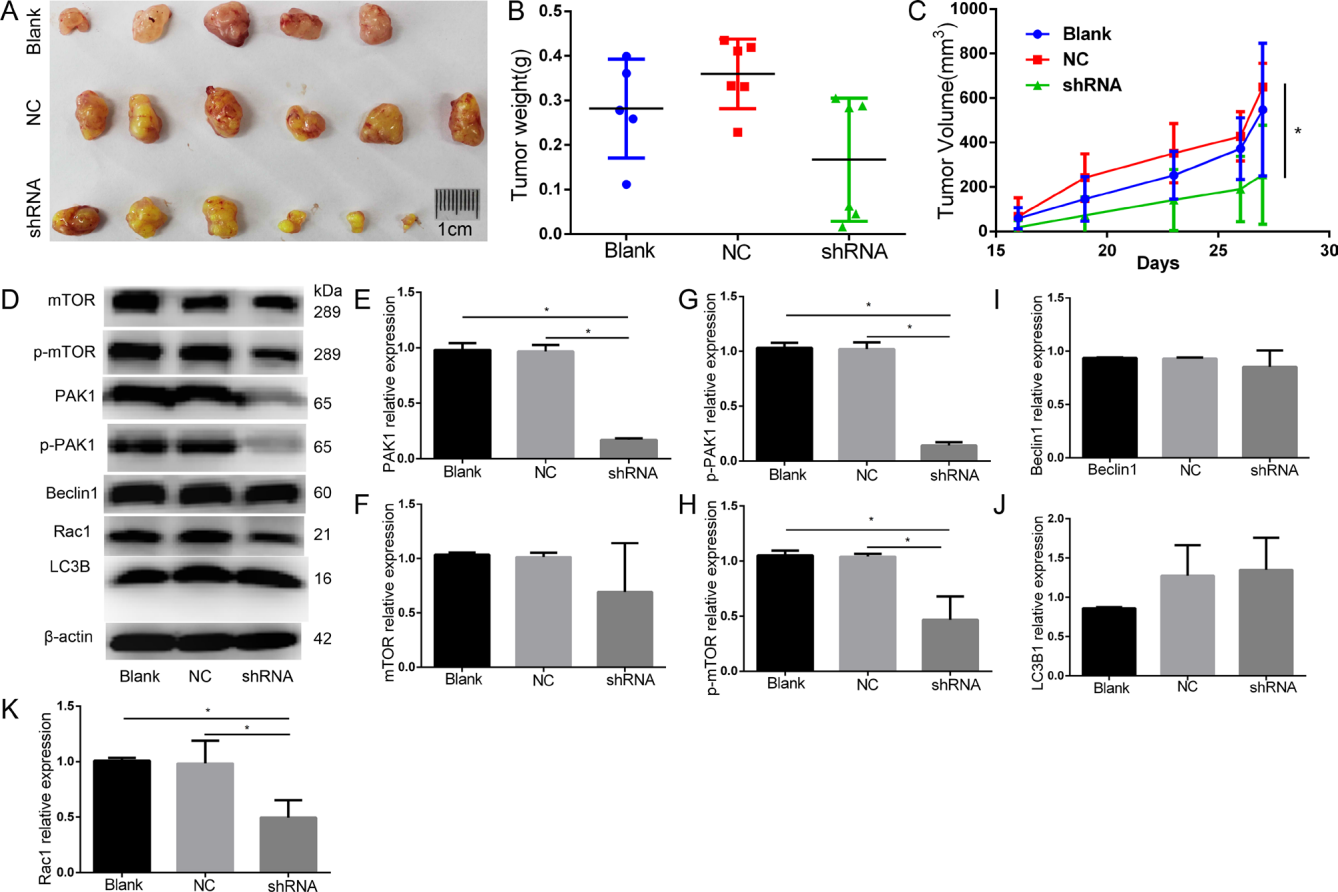
Animal study knockdown of PAK1 decreased DU145 PCa xenograft growth by compared with the negative control (**A**) The original tumor. (**B**) Tumor weight(g). (**C**) Tumor volume(mm^3^), tumor volume of shRNA was smaller than that of NC on day 19, 23, 26, 27. (**D**) Western blot. (**E**) PAK1 expression decreased in PAK1-shRNA expressing cells than Blank and NC. (**F**) mTOR relative expression. (**G**) p-PAK1 expression decreased in PAK1-shRNA expressing cells than Blank and NC. (**H**) p-mTOR expression decreased in PAK1-shRNA expressing cells than Blank and NC. (**I**) Beclin1 relative expression. (**J**) LC3B1 relative expression. (**K**) Rac1 expression decreased in PAK1-shRNA expressing cells than Blank and NC.*P < 0.05.

Normal cells, negative control and PAK1-shRNA-expressing cells of DU145 cells were engrafted onto the nude mice (6 mice per group) to monitor tumor growth, respectively. Tumor volume of shRNA was smaller than that of NC on day 19, 23, 26, 27, and tumor weight of shRNA tended to be lighter than Blank and NC, and knockdown of PAK1 decreased DU145 PCa xenograft growth compared with the negative control. The expression of PAK1 in tumor of shRNA was significantly decreased both at mRNA and protein levels in these of Blank and NC (Figure 8).

## DISCUSSION

The current study showed the overexpression of PAK1 in human PCa and evaluated the PAK1 expression associated with clinicopathological features of patients with PCa, and provided the evidence regarding elucidation of potential mechanisms of mTOR inhibitor rapamycin and activator MYH1485 chemotherapy. In our retrospective study, we revealed that PSA and AKP were notable higher in PCa patients than that in BPH patients, and it has come to our attention that larger prostate size, more scleroid texture and more tubercle existed, and more central sulcus disappeared during digital rectal examination in PCa, further, more abnormal echo, lesion and signal existed in ultrasonography, computed tomography and magnetic resonance imaging (MRI), respectively. Higher PSA and AKP and lower haematoglobin were detected in metastasis PCa when comparing with non metastasis PCa, and touched scleroid prostate and the PSA more than 50 ng/ml were related to positive PAK1 expression in PCa. Flexible texture and higher haematoglobin were detected in BPH with negative PAK1 expression. According to immunohistochemistry results, higher positive expression of PAK1, p-PAK1, Ki-67, P504s and P53 was shown in PCa, both P63 and CK5/6 expression was negative in PCa, and higher positive expression of 34βE12, P63 and CK5/6 was in BPH, but the difference of mTOR, p-mTOR, PSA, PAP, AR and CK expression was not statistically significant between PCa and BPH. CK20 expression in PCa tends to be in accordance with BPH, while CK7, vimentin and SMA tend to be negative in PCa and positive in BPH.

Michael D. Bright et al. [17]have found that PAK1 protein expression in DU145 is higher than PC-3, and knockdown of PAK1 in DU145 inhibits HGF-stimulated migration and loss of cell–cell junctions, whereas knockdown of PAK1 in PC3 reduces HGF-stimulated migration. Anna Goc, et al. [18] have indicated that PAK1 expression in PC-3, C4-2 and VCaP was higher than LNCaP, and PAK1 expression was elevated significantly in PCa and metastatic lymph node and lung tissues, the study reported that PAK1 knockdown impaired PCa growth via increased expression of TGF-β and reduced secretion of MMP9, and then Ahmad Al-Azayzih, et al. [19] have researched DU145 and PC-3 and demonstrated that TGF-β1 induces apoptosis and EMT in the cells via activation of P38-MAPK and Rac1/Pak1 respectively. Yong Jae Shin, et al. [20] have shown that growth of PC-3 is inhibited by the treatment of a PAK1-inhibiting peptide comprising 19 amino acids centered on S79, but not by the PAK1 peptide containing the S79A mutation. In addition, Yong-Bae Kim et al. [21] have analyzed PC-3 and RWPE-1 and demonstrated that PI3K activates PAK1 at the plasma membrane by promoting CK2 phosphorylation of PAK1 via CKIP-1.

Vineeta Khare [22] have demonstrated that PAK1 expression is regulated by MEK, PI3K, and mTOR in normal diploid colon epithelial cells (HCEC-1CT) and PAK1 and beta-catenin expression correlated and inhibition of PAK1 and addition of 5-ASA elicited similar molecular affects by reducing ERK and AKT activation in colorectal cancer cell lines. MTOR is constitutively activated in various types of human cancers development [23], and mTOR signaling networks have emerged as attractive targets for novel anticancer therapies [24].

The researchers exploring PAK1 and PCa have finished excellent work, but lack the human BPH and adequate PCa tissues studies. Our previous research [25] have demonstrated that the overexpression of PAK1 is closely associated with the clinicopathological features of bladder cancer (BC), and PAK1 may play an important role in the development and progression of BC, accordingly, we implemented our PCa and BPH patients clinical analysis, and four kinds of cells including benign prostate epithelial cells RWPE-1, metastatic androgen independent human prostate cancer DU145 and PC-3 cells and androgen dependent prostate cancer cells LNCaP were tested with the treatment of mTOR inhibitor-rapamycin and activator-MYH1485, respectively.

Our study manifested that PAK1 expression were increased in prostate cancer tissues compared with BPH, and PAK1 mRNA and protein in DU145 and PC-3 were obviously higher than LNCaP and RWPE-1, respectively, in addition, PAK1 expression was increased in DU145 compared with PC-3. The Phosphoinositide 3-kinase (PI3K)-AKT mammalian target of rapamycin (mTOR) pathway [26], and mTOR pathway is up-regulated in castration-resistant prostate cancer (CRPC) [27]. MTOR signaling is related to the regulation of apoptosis, autophagy, neurogenesis, angiogenesis, et al, and mTOR can suppress autophagy, and mTOR inhibitors can strongly induce autophagy [28], and autophagy is impaired through the activation of PI3K–mTOR signaling [29], and autophagy inhibition enhances susceptibility to oxidative damage and apoptosis, whereas the activation cause apoptosis inhibition [30]. Moreover, mTOR can be phosphorylated at serine 2448 in PCa, and mTOR inhibitor can block mTOR signaling while MHY1485 can increase the phosphorylation mTOR level. Based on the article about rapamycin and MYH1485 effects on cells [31–33], and we previously reported that different concentrations of inhibitor rapamycin remarkably inhibited PC-3 cell proliferation after 48 h (*P* < 0.05), inhibitory action did not change significantly from 5–100 nM [34], then we used rapamycin and MHY1485 to investigate the molecular mechanisms of PAK1 impact on PCa cells.

Though some researchers demonstrated that allosteric mTOR inhibitors had limited clinical efficacy in advanced PCa clinical trials [35, 36]. Rapamycin in combination with other compound or inhibitors had enhanced efficacy against prostate cancer [37] or led to decreased cell proliferation [38], and Giovanni Luca Gravina work also suggested that PI3K/Akt/mTOR inhibitors can be used in hormone-insensitive prostate cancer models [39].

We investigated PAK1, p-PAK1, mTOR, p-mTOR, Beclin1, LC3B expression in DU145 after 12h with different concentrations of rapamycin and MHY1485 treatment. We observed that the majority of DU145 died with 100 nmol rapamycin and all cells died with 1000 nmol rapamycin after 24 h, then we use 10 nmol rapamycin for further research. PAK1, p-mTOR and LC3B1 tended to increase with 0.1 μmol MYH1485 after 12 h.

In DU145, PAK1, p-PAK1, mTOR and LC3B1 expression increased more with MYH1485 than rapamycin. P-mTOR expression decreased with rapamycin treatment in DU145, while increased with MYH1485, and Becin1 expression can increase in cells with rapamycin. In LNCaP, PAK1, Becin1 and LC3B3/LC3B1 ratio can increase with rapamycin treatment. p-mTOR expression increased with MYH1485 treatment and decreased with rapamycin treatment

It may indicate that PAK1 and p-PAK1 were regulated by mTOR with different process, and Beclin1 and LC3B expression may be regulated by PAK1 or mTOR through autophagy process. To further study PAK1 on biological behaviors and mechanisms on PCa cells, we knocked down PAK1 expression in DU145 using shRNA approach, we found that Knocking down of PAK1 resulted in a reduction of cell proliferation after 72h, and leaded to a decreased of migration, and invasion. Expression of p-PAK1, mTOR and Beclin1 decreased along with PAK1, and LC3B1 of shRNA cells decreased than the blank and NC cells, meanwhile, LC3B2/LC3B1 ratio of shRNA cells tended to diminish, which indicated that autophagy degraded, suggesting that PAK1 may involved in autophagy in DU145 cell lines.

Knockdown of PAK1 decreased DU145 PCa xenograft growth in nude mice by compared with the negative control, Western blot analysis suggested that PAK1, p-PAK1, Rac1 and p-mTOR expression was decreased in shRNA group than the NC and Blank, and mTOR expression tended to be decreased, indicating that PAK1 may promote the progression.

Shuchen Gu et al [40] found inhibition of mTOR blocked p70S6K and PAK1 phosphorylation and actin remodeling and demonstrated a mAR-governed pathway involving FAK/PI-3K and mTOR/p70S6K/PAK1-cascade that regulates early actin reorganization in colon cancer cells. Roberta L. Beauchamp et al. [41] reported that independent activation of SGK1 and PAK1 may be partly responsible for the mTORC1 activation in NF2-deficient meningioma cells, and the group I PAK inhibitor FRAX597 may be improper for treatment for higher concentrations needed. Treatment with specific inhibitors of growth signaling pathways (MEK/PI3K/mTOR) demonstrated that in normal diploid colon epithelial cells (HCEC-1CT), PAK1 expression is regulated by MEK, PI3K, and mTOR [42]. Low mTOR activity in DM T cells and as mimicked by Rapamycin, Rapalog, KU, and Raptor siRNA treatment increased PAK1 signaling whereas mTOR activation by treatment with leucine, non-essential amino acids and pyruvate decreased PAK1 signaling [43].

Our data indicated that, PAK1 was related with the clinicopathological features of PCa patients, and PAK1 may be a diagnostic factor in PCa. PAK1 could be further investigated as a potential therapeutic target of prostate cancer.

## MATERIALS AND METHODS

### Patients and samples

The study on human subjects was approved by the Ethics Committee of Jinshan Hospital, Fudan University. The human samples were obtained from biopsies or surgical specimens of patients with PCa and benign prostate hyperplasia (BPH). The histological grades and clinical stages of all specimens were judged and classified by experienced surgeons and pathologists based on the World Health Organization (WHO) classification and the tumor node, and metastasis (TNM) staging system from the American Joint Committee on Cancer (AJCC). All patients were married men of the Chinese Han Nationality. No patients had received adjuvant chemotherapy or radiotherapy before operation, and 113 cases confirmed by surgery and histopathology were retrieved for this study. A total of 40 paraffin-embedded samples from 40 BPH patients (median age 68.5 years, range 53–84) were collected, while 73 PCa samples from 73 prostate cancer patients (median age 73.4 years, range 56–89) at Jinshan Hospital, Fudan University from 2007 to 2015. Among the patients diagnosed with PCa, 31 patients progressed but survive and 10 patients died, while among the BPH patients, 23 patients recovered and 1 patient died, upon to March 2016.

### Immunohistochemistry

Immunohistochemical staining was done from paraffin-embedded tissue sections to evaluate the expression of PAK1 protein in PCa and BPH specimens. A formalin-soaked, paraffin-encapsulated tissue block was cut into 4 μm-thick sections, which were placed on slides. Some of the slides were stained with standard haematoxylin and eosin (H&E). For immunohistochemistry (IHC), the section was heated at 60°C for 2 h, then dewaxed in xylene and rehydrated in decreasing concentrations of alcohol. To restore the antigen, the slides were placed in 0.01 mol/L citrate buffer (pH 6.0), while antigen retrieval was performed using Tris-EDTA buffer (PH 9.0) only when anti-phosphor-mTOR (S2448) antibody was detected, and heated in the microwave oven (100°C) for 2 minutes. The treated slides were rinsed with phosphate buffered saline (PBS) three times for 5 minutes. Endogenous peroxidase activity was blocked by placing the slides in 3% hydrogen peroxide for 15 minutes at room temperature, followed by three times washes for 5 minutes with PBS. After blocking with 10% normal goat serum (Maixin Bio, Fuzhou, China) for 20 minutes at room temperature, the section was incubated with rabbit anti-PAK1 antibody (1:500 dilution, Cell Signaling Technology, Beverly, MA, USA), anti-phospho-PAK1 antibody (1:300 dilution, Phospho T212, Abcam, Cambridge, MA, USA), mTOR (7C10) Rabbit mAb (1:60 dilution, Cell Signaling Technology, Beverly, MA, USA), anti-mTOR (phosphor S2448) antibody(1:50 dilution, Abcam, Cambridge, MA, USA) at 4°C overnight, respectively, and then incubated with biotinylated anti-rabbit secondary antibody (Maixin Bio) at room temperature for 1 h. After washing three times for 5 minutes with PBS, the signal was detected by DAB Kit (diaminobenzidine, Maixin Bio). The negative control consisted of replacing the primary antibody with PBS.

Scoring of immunoreactive staining was performed by two independent pathologists without any prior knowledge of patient's clinical data. The proportion of positive cells was scored by the extent of immunoreactive staining to the following categories, the percentage of positive staining was scored as 0 (0%, no positive cells), 1 (≤ 25% positive cells), 2 (26%–50% positive cells), 3 (51%–75%positive cells), or 4 (> 75% positive cells). The intensity of immunostaining was scored as 0 (no positive staining), 1 (weakly stained), 2 (moderately stained), or 3 (strongly stained). A final immunoreactive score was determined by the sum of the positive proportion and the staining intensity. The final score was clustered into four groups: -, ≤ 2 total points; +, 3–4 total points; ++, 5–6 total points; and +++, 7 total points. In this study, - and + represent no or low expression, whereas ++ and +++ indicate high expression. Finally, for this study, we defined the cases with grades equal to 0 and 1 as PAK1 negativity and those with grades equal to 2 and 3 as PAK1 positivity.

### Cell lines and cell culture

Cells were obtained from Shanghai Cell Bank of Chinese Academy of Science (Shanghai, China) and cultured without penicillin or streptomycin at 37°C in a humidified atmosphere with 5% CO_2_. The benign prostate epithelial RWPE-1 cells were cultured in keratinocyte serum-free medium (K-SFM) containing 5 ng/ml of epidermal growth factor (EGF) and 0.05 mg/ml of bovine pituitary extract (BPE) without fetal bovine serum. Metastatic androgen independent human prostate cancer PC-3 cells were maintained in F12K medium with 10% fetal bovine serum (FBS, GIBCO, Life Technologies, Australia), while DU145 cells in MEM basic medium with 5% sodium pyruvate, 5% MEM Non-Essential Amino Acids and 10% FBS. Androgen dependent prostate cancer LNCaP clone FGC (LNCaP) cells were maintained in RPMI-1640 medium supplemented with 10% FBS.

### Cell treatment

Cells were plated in 6-well plates at a total volume of 2 ml per well, and rapamycin and MYH1485 were reconstituted in DMSO to the suitable stock concentration and diluted shortly before the experiments to the final concentrations depicted in the Figures. DU145 cell lines were incubated with various concentrations of rapamycin (1, 10, 100 and 1000 nmol/L) and MYH1485 (0.1, 1, 10 and 100 μmol/L) for 12h at 37°C. DU145, LNCaP and RWPE-1 Cells were detected at 1, 6, 12, 24 and 48 h with rapamycin (10 nmol/L) and MYH1485 (0.1 μmol/L), respectively.

### RNA extraction and quantitative real-time PCR

Total RNA was isolated from cell culture using an RNA extraction kit (Axygen BIOSCIENCES, Corning, USA) according to the manufacturer's protocol and reversely transcribed into cDNA using the reverse transcription kit (Takara Biotechnology CoLtd., Dalian, Liaoning, China). The primer sequences were: human PAK1 forward, 5′-AGTTTCAGAAGATGAGGATGATGA-3′; human PAK1 reverse, 5′-AATCACAGACCGTGTGTATACA G-3′, human β-actin forward, 5′-ACAATGTGGCCGA GGACTTT-3′; human β-actin reverse, 5′-GCACGAAGGC TCATCATTCA-3′ (synthesized by Sangon Biotech Co., Ltd., Shanghai, China).

An initial step of denaturing RNA at 95°C for 30 seconds was applied, then PCR amplification was performed at 95°C for 5 seconds and 60°C for 31 seconds for 40 cycles in a 20 μl reaction system with SYBR Select Master Mix (Invitrogen) using the ABI PRISM 7300 Real-Time PCR system (Applied Biosystems, Foster City, CA, USA). All reactions were in triplicate and repeated three times. For relative quantification, target (PAK1) was normalized to an endogenous control (β-actin) given by 2^ΔΔCt^.

### Western blot analysis

Cells were washed with PBS and harvested using RIPA Buffer (Beyotime) with 1% PMSF(BIOTECH WELL) and phosphatase inhibitors (KeyGEN BioTECH), followed by sonication. All the lysates were cleared by centrifugation (15,000 rpm) at 4°C for 20 minutes. The protein concentrations were measured using the BCA protein assay kit (Thermo SCITIFIC). A total 25 μg of protein from cell lysate was separated on 6% or 10% SDS-PAGE gel and then transferred to polyvinylidene difluoride membrane (PVDF, Millipore, Billerica, MA, USA). After blocking with 5% non-fat milk in Tris-buffered saline with Tween-20 (TBST) for 1 h, the membrane was incubated with primary antibody at 4°C overnight. The following primary antibodies were used for analysis: rabbit anti-PAK1 (1:1000 dilution, Cell Signaling Technology), anti-phospho-PAK1 (1:1000 dilution, Phospho T212, Abcam), mTOR (7C10) Rabbit mAb (1:1000 dilution, Cell Signaling Technology, Beverly, MA, USA), anti- phosphor-mTOR (S2448) antibody (1:1000 dilution, Abcam, Cambridge, MA, USA), LC3B(1:1000 dilution, Cell Signaling Technology), Beclin1 (1:1000 dilution, Cell Signaling Technology). After washing with TBST for three times for 10 min, the membrane was subsequently incubated with horseradish peroxidase-conjugated goat anti-rabbit IgG second antibody (1:5000 dilution, GENTAUR, USA). Loading equivalency was determined using a mouse beta actin monoclonal antibody (1:5000 dilution, Proteintech, People's Republic of China), and the second antibody was goat anti-mouse IgG, Peroxidase conjugated, H+L(1:5000 dilution, Millipore, USA). Signals were detected using Immobilon™ Western Chemiluminescent HRP Substrate (Millipore) and quantified using Tanon-4500 Gel Imaging System with GIS ID Analysis Software v4.1.5 (Tanon Science & Technology Co., Ltd., Shanghai, China).

### Generation of PAK1-shRNA and lentiviral transduction

Human PAK1 short hairpin RNA (PAK1-shRNA) was constructed with double-strand oligonucleotides corresponding to the target sequence of GATGCT TTGACCCGGAATA and inserted into pHY-LV-KD5.1 RNAi lentivirus (Hanyin Biotechnology Co., Ltd., Shanghai, China). The top strand of PAK1-shRNA is 5′-gatccGATGCTTTGACCCGGAATATTCAAGAGATA TTCCGGGTCAAAGCATCTTTTTTg-3′, and the bottom strand of PAK1-shRNA is 5′-aattcAAAAAAGATGCTTT GACCCGGAATATCTCTTGAATATTCCGGGTCAAAG CATCg-3′. A control shRNA (negative control, NC) was also supplied by Hanyin Biotechnology. For the generation of PAK1-shRNA-expressing cells and their counterpart control cells, normal DU145 (Blank) cells were plated in six-well plate for 24 h and then infected with PAK1-shRNA or control shRNA lentiviral particles at the concentration of 10 MOI for 48 h. The efficiency of PAK1-shRNA lentiviral transduction was examined by fluorescence microscopy as the constructs contained green fluorescent protein (GFP). Knocking down of PAK1 at messenger RNA (mRNA) and protein levels was confirmed using PCR and Western blot as indicated above. The fourth or fifth passaged cells that stably expressed PAK1-shRNA were used for cellular assays described below.

### Cell proliferation assay

Blank, NC and PAK1-shRNA-expressing cells were plated into 96-well plate at a density of 4 × 10^3^/well, respectively, and cultured for 24, 48 and 72 h. Cell proliferation was measured using the Cell Proliferation Reagent (WST-1) kit (Roche, Cat#11644807001) according to the manufacturer's instruction. The signal in OD was read at 450 nm by a plate reader (BioTek Epoch, Winooski, VT, USA). Experiment was repeated at least three times.

### Wound healing assay

Cells were seeded into 6-well plates at a density of 3.5 × 10^5^ cells/well and cultured up to 85% confluence. The cell monolayer was then scraped using a pipette tip to generate scratch wounds. After washing three times to remove cell debris, the cells were incubated with serum free MEM medium for 24, 48 and 72 h. Images were obtained at different time points and the widths of the gaps were measured and analyzed.

### Cell invasion assay

The Matrigel (250 μg/ml final concentration, BD Biosciences, Cat# 356234, Bedford, MA, USA) was coated on the top chamber of Transwell (pore size 8 μm, Costar, Corning, Cat# 3422, New York, NY, USA) in a 24-well plate. After the solidification of the Matrigel, control and PAK1-shRNA-expressing cells were plated into the upper chamber of Transwell at a density of 10 × 10^4^ cells/well with 100 μl of serum free MEM medium for 48 h, respectively. The lower chamber of 24-well plate was filled with 700 μl of MEM medium containing 10% FBS. The upper surface of chamber was softly wiped with the cotton swab and the lower surface with migrated cells was fixed by 4% paraformaldehyde for 20 minutes and stained with 0.1% crystal violet for 25 minutes. Stained cells were photographed, and cell number was counted in five random fields under a light microscope. All experiments were repeated three times.

### Colony formation assay

A cell suspension (200 cells) in 2 ml MEM medium supplemented with 10% FBS were layered onto 6-cm plates. Plating was carried out in triplicate and repeated at least three times. After 14 days of growth, cells were washed twice with PBS, fixed with 4% paraformaldehyde for 20 min, and then stained with 0.1% crystal violet for 25min. And colonies were photographed and reported.

### Animal study

Six-week-old male nude mice from were housed on a 12 h light–dark cycle with access to food and water ad libitum. Normal DU145 cells (Blank), negative control (NC) and PAK1-shRNA-expressing (shRNA) cells of DU145 (1 × 10^6^ cells) were inoculated subcutaneously into the oxter of mice (3 groups, and 6 mice per group). Tumor size and the body weights of mice were also recorded twice a week and volumes were determined using the formula volume = length × width^2^ /2. The body weights of mice were also recorded twice a week. Twenty seven days after tumor inoculation, tumor tissue was excised from the mice and weighed until the tumor length of NC group was more than 10 millimeter (mm), then western blot analysis, quantitative real-time PCR and IHC were operated.

### Statistical analyses

Statistical analyses were performed using SPSS software version 18.0 (SPSS, Chicago, IL, USA). Data are presented as the mean ± standard deviation (SD). The association between PAK1, p-PAK1, mTOR, et al protein expression and pathological parameters of PCa and BPH was performed with chi-square tests. For analyzing experimental data of real-time PCR, Western blot, and cellular assays, a Student's *t* test was used. Overall survival (OS) was defined as the interval from date of diagnosis until death from any cause. The patients were censored if they were still alive or the patients lost to follow-up until the last follow-up. We assessed OS using a Kaplan-Meier method. A *P* value < 0.05 was considered to be significant.
